# Randomized controlled trial of clopidogrel to prevent primary arteriovenous fistula failure in hemodialysis patients

**DOI:** 10.4103/0971-4065.53323

**Published:** 2009-04

**Authors:** A. Ghorbani, M. Aalamshah, H. Shahbazian, A. Ehsanpour, A. Aref

**Affiliations:** Department of Nephrology, Dialysis and Kidney Transplanation, Jondi Shapour University of Medical Sciences, Ahwaz, Iran

**Keywords:** Arteriovenous fistula, clopidogrel, hemodialysis, primary AVF failure, vascular surgical procedures

## Abstract

The optimal vascular access for chronic maintenance hemodialysis is the arteriovenous fistula (AVF). Several studies suggest a role for antiplatelet agents in the prevention of primary AVF failure. A double-blind, randomized trial was conducted to assess the efficacy and safety of clopidogrel in hemodialysis patients. Ninety three patients were randomized to receive 75 mg/daily of clopidogrel or placebo. The treatment was initiated 7–10 days prior to scheduled access surgery and continued up to six weeks postoperatively, and then patients were monitored for six months. The primary outcome was AVF failure eight weeks after fistula creation. With a permuted block randomization schedule, 46 patients received clopidogrel and 47 patients received control placebo. The primary AVF failures at two months were 21.6% in placebo group and 5.2% in clopidogrel group (*P* = 0.03). The hazard ratio for the incidence of primary AVF failure was 0.72 (CI 95%, 0.41–1.01). Analysis of covariables indicated that this effect occurred principally as a result of clopidogrel administration. First hemodialysis from newly created AVF in clopidogrel group was significantly more successful than placebo group (*P* = 0.008). No life-threatening adverse event or severe bleeding was recorded in both groups. Clopidogrel seems to be effective and safe for prevention of primary AVF failure in hemodialysis patients.

## Introduction

Performance of a successful hemodialysis procedure requires a functional vascular access. The preferred type of access is a native fistula because they have the lowest risk of complications, lowest need for intervention, and the best long-term patency.[1] Once an arteriovenous fistula (AVF) is created, it must develop to the point that it is usable. Vascular access dysfunction is one of the most important causes of morbidity in the hemodialysis population.[2] Primary failure of native fistulas occurs as a result of either thrombosis within the first several weeks following surgical creation (early thrombosis), or inadequate maturation of the vein.[3] The primary AVF failure rate is approximately 9–50%.[4–6] Fistula evaluation 4–6 weeks after creation should be considered mandatory.[7] The clinical manifestations of early fistula failure are failure to develop adequately to permit repetitive cannulation for dialysis, inadequate flow to support dialysis, and thrombosis. The characteristic pathology that results in AVF failure is a juxta-anastomotic stenosis.[8] Whether primary AVF failure can be prevented with pharmacologic agents has not been extensively examined. Several studies have indicated that the frequency of AVF failure and loss can be reduced with antiplatelet agents.[9–18] Although those results are encouraging, they do not provide conclusive evidence of the efficacy of antiplatelet agents among patients with AVF. On the basis of these considerations, we performed a randomized, double-blind trial to test the hypothesis that clopidogrel, would prevent primary AVF failure among hemodialysis patients.

## Materials and Methods

The study was a randomized, double-blind trial. Patients of minimum age 18 years close to the initiation of chronic hemodialysis requiring AVF, and patients who were undergoing chronic hemodialysis but requiring a new AVF at a different site were the inclusion criteria. Exclusion criteria included patients with a history of gastrointestinal bleeding or previous bleeding episodes within six months prior to initiation of the study, patients already receiving chronic anticoagulation therapy (antiplatelet agents or warfarin), patients with terminal or life-threatening disease, pregnancy, malignant hypertension, a platelet count of <100,000/mm^3^, and other demonstrated medical conditions that would make antiplatelet therapy dangerous. All patients were recruited from the outpatient hemodialysis program at Jondi Shapour University, and the same surgical team placed all fistulas.

Randomization was performed centrally, by the coordinating center. The randomization was stratified according to medical center with a permuted block scheme, with a block size of four and equal allocation. After identifying and obtaining consent from eligible participants, the local study coordinator telephoned the coordinating center to obtain a randomization number, which corresponded to a specific medication bottle available in the local research pharmacy. Neither the details of the randomization sequence nor the identity of the medication assignment was known to the participant or any personnel at the participating sites. Consenting eligible participants were randomized to receive either clopidogrel (75 mg/daily) or matching placebo (47 patients in placebo group and 46 patients in clopidogrel group). The treatment was initiated 7–10 days prior to scheduled access surgery and continued up to six weeks postoperatively, with full approval by the Jondi Shapour University Institutional Review Board. Patients were monitored for occurrence of complications, need for hemodialysis until six months after trial.

The primary null hypothesis of the study was that clopidogrel would have no preventive effect on the incidence of primary AVF failure. The primary outcome was AVF failure eight weeks after fistula creation. Fistula failure was determined by a member of the study team (either the study coordinator or the site principal investigator), who was blinded to the treatment allocation. The fistula was classified as patent if a bruit was detectable along the vein at least 8 cm proximal to the arteriovenous anastomosis throughout systole and diastole. Secondary outcomes included adverse events and mortality. Platelet hemostatic function was measured monthly, as whole-blood bleeding time. Routine blood chemistry profiles, dialysis prescriptions, body weights, medications, and complications for all patients were recorded in a computerized database, and thus were available for inclusion in the final data analysis. We obtained detailed information on bleeding events. Assessment of the severity of bleeding episodes was performed by a panel blinded to the treatment assignments. Discontinuation of the study drug following any bleeding event was the rule.

### Statistical analysis

Data are presented as mean ± SE for continuous variables and as percentages for categorical variables. All data were tested for normality using the method of Kolmogorov-Smirnov. Statistical analyses were performed on an intention-to-treat basis. The *t* test was used when the means of two groups were compared. On the basis of intention-to-treat principles, all other participants for whom study medications were discontinued continued to be monitored according to the protocol. All hypothesis tests were conducted by using a significance level of 0.05 (two-sided). The statistical program SPSS version 13 (SPSS, Chicago, IL) was used to analyze the data.

## Results

Between December 2006 and March 2008, a total of 93 patients met the study criteria for enrollment. The demographic and baseline laboratory findings for each group are summarized in Table 1. There were no significant differences in covariables between groups. None of the covariables (covariables thought to influence the risk of AVF included age, gender, diabetes mellitus, bleeding time, and blood pressure) measured at baseline or follow-up times was correlated with the development of AVF failure. All patients were taking study medication 7–10 days prior to surgery. However, the medication had to be discontinued prematurely in 18 (19.4%) patients because of no intention to complete trial by six cases and complication occurring in 11 cases. One patient died before the end of trial. Two patients in clopidogrel group and three patients in placebo group were enrolled after creation of a second fistula. Prior AVFs failed because of late fistula failure in all of them. Finally, 75 patients completed trial (38 patients in clopidogrel and 37 patients in placebo groups). None of the patients was lost to follow-up.

**Table 1:** Patients characteristics at baseline

Characteristic	All patients (%)	Clopidogrel (%)	Placebo (%)	*P* value
Age (years)	45.03 ± 1.2	44.23 ± 3.36	45.8 ± 2.84	0.47
Males	48 (51.6)	24 (25.8)	24 (25.8)	0.91
Diabetes mellitus	25 (26.9)	14 (15.1)	11 (11.8)	0.44
Patients on hemodialysis	63 (67.7)	29 (31.2)	34 (36.6)	0.33
Drug use by the end of trial	75 (80.6)	38 (40.9)	37 (39.8)	0.63

Five and three fistulae in clopidogrel-treated and placebo groups were proximal AVFs. Two patients in clopidogrel-treated group (5.26%) showed an early failure of the AVF compared to eight patients (21.62%) in placebo group (*P* < 0.05) [Table 2]. There was significant benefit of active treatment in the prevention of AVF failure. The hazard ratio for the incidence of primary AVF failure was 0.72 (CI 95%, 0.41–1.01). Fistula locations had no effects on the development of AVF failure in either group. During follow-up from eight weeks to six months no important complication occurred. Sixty patients required hemodialysis during this time. Hemodialysis was tried in 26 patients of clopidogrel group within six months after AVF creation and it was successful in 24 patients (92.3%). In placebo group, hemodialysis from new AVF was tried in 34 patients and it was successful in 24 cases (70.5%). First hemodialysis from newly created AVF in clopidogrel group was significantly more successful than placebo group (*P* = 0.008). The cumulative incidence of bleeding was similar between groups [Table 3]. No severe bleeding episode such as intracranial hemorrhage or life-threatening bleeding was recorded during active treatment period. There were no deaths attributable to bleeding in either treatment group. Bleeding times were similar at baseline for clopidogrel group (8.1 ± 0.3 min) and placebo group (8.4 ± 0.6 min) and remained stable (8.5 ± 0.4 min for clopidogrel group and 8.6 ± 0.3 min for placebo group) throughout the study period (*P* = 0.21). In addition, there were no differences between baseline and follow-up hematocrit values or changes in recombinant human erythropoietin doses during the study period for either group.

**Table 2:** Clinical outcomes at the end of trial

Outcome		All patients (%)	Clopidogrel (%)	Placebo (%)	*P* value
Primary AVF failure	Drug use by the end of trial	10 (26.88)	2 (5.26)	8 (21.62)	0.03
	Premature cession of drug	2 (2.15)	0 (0)	2 (2.15)	

**Table 3:** Complications and mortality

Complication	All patients (%)	Clopidogrel (%)	Placebo (%)	*P* value
GI bleeding	5 (5.3)	2 (2.1)	3 (3.2)	0.31
Non GI tract bleeding	9 (9.6)	5 (5.3)	4 (4.3)	0.63
Death events	4 (4.3)	2 (2.1)	2 (2.1)	0.47

## Discussion

Chronic maintenance hemodialysis requires stable and repetitive access to the intravascular compartment in order to deliver high rates of blood flow to the extracorporeal circuit. The AVF is the method of choice for the establishment of hemodialysis vascular access in patients with endstage renal disease.[1] The fistula is relatively simple to perform under local anesthesia and, when successfully established, is easy to needle and relatively free from complications. However, a significant proportion (9–50%) of fistulas fails early within three months of surgery.[4–6] An AVF with primary failure is defined as a fistula that never provided reliable hemodialysis.[18] Vascular access failure is the most common reason for hospitalization among hemodialysis patients.[19] The typical lesion of access thrombosis is neointimal vascular smooth muscle cell proliferation in the anastomotic draining vein. Platelet activation from endothelial injury may play an important role in stimulating platelet aggregators such as PDGF and thromboxane A2, in addition to directly stimulating vascular intimal proliferation.[18] Therefore, the therapeutic potential of antiplatelet agents including aspirin, sulfinpyrazone, dipyridamole, and ticlopidine were tested.[9–17] Our study was undertaken to determine the effects of clopidogrel on the incidence of primary AVF failure among newly created AVFs. We observed a significant risk reduction in the primary AVF failure in active treatment group compared to placebo group. The results of our analysis suggest that daily administration of 75 mg of clopidogrel, beginning 7–10 days prior to AVF creation, was successful in preventing the development of vascular failure with acceptable side effects. We were unable to account for the differences observed in our clinical trial on the basis of age, gender, diabetes mellitus, bleeding times, hematocrit levels, or weekly doses of recombinant human erythropoietin. This finding suggests that the risk reduction in vascular failure might be attributed to clopidogrel administration. Our results are supported by recent Cochrane report.[20] This meta-analysis confirmed the beneficial effect of antiplatelet treatment as an adjuvant to increase the patency of AVFs in the short term. However, there have been multiple studies showing variable results of antiplatelet agents on vascular access failure. Yevzlin *et al*., showed a negative association between antiplatelet therapy and access patency.[21] In this trial, usage of some drugs was not associated with significant risk reduction in access failure. Moreover, antiplatelet therapy in patients with access failure was associated with significantly increased risk of access failure. Kaufman *et al*., demonstrated no change in the risk of graft thrombosis with aspirin plus clopidogrel therapy.[22] They also noted that in chronic hemodialysis patients there is a trend toward increased thrombosis with aspirin therapy. Also Kooistra *et al*., were unable to demonstrate a benefit with low-dose aspirin on thrombovascular events in 68 hemodialysis patients.[23] In contrast, combining all the studies of antiplatelet agents in patients with new primary fistulae in which there was a placebo control group, the thrombosis rate in the control group was significantly higher than active treatment group.[20] Three trials compared ticlopidine with placebo with a total number of 312 participants undergoing AVF formation or graft interposition. All three trials comparing ticlopidine with placebo favored treatment in both AVF and vascular grafts. In the Fiskerstrand study, two out of six patients in the ticlopidine group compared with five out of nine in the placebo group, developed fistulae thromboses at one month (OR = 0.40, CI 95%, 0.05– 3.42).[15] In the earlier Grontoft study, only two out of 19 who received treatment developed fistulae thromboses compared to eight out of 17 on placebo (OR = 0.13, CI 95%, 0.02–0.76).[13] In Gontoft 1998, 16 out of 130 patients who received ticlopidine developed thromboses in the fistulae compared with 25 out of 131 in the placebo group (OR = 0.60, CI 95%, 0.30–1.18).[14] The overall result of the meta-analysis also favored treatment (OR = 0.47, CI 95%, 0.26–0.85). The overall *P*-value was 0.01.[20] We also assessed the effect of clopidogrel on the successful initiation of hemodialysis via AVF. The rate of the performing successful first hemodialysis via AVF was greater in clopidogrel group.

The overall incidence of bleeding events was 15% in our study. According to Kaufman *et al*., we expected to encounter with 16 episodes of bleeding during six months in our patients.[22] However, the incidence of bleeding episodes was lower than expected. This finding might be related to restrictive exclusion criteria.

This study is the first carefully monitored trial that was limited to AVF. It is a well-known fact that AVFs have lower thrombosis rates compared with AVF grafts. In multiple studies, AVFs have been shown to have significantly improved patency rates and lower complication and infection rates. Some studies pointed out that antiplatelet therapy might be more effective in fistulas than in arteriovenous grafts.[14,22] We studied this hypotheses for first time. Our results suggested that clopidogrel is an effective therapy in prevention of primary AVF failure.

The major limitation of our study is the small number of patients, but in view of the promising results we believe that our preliminary findings deserved prompt communication. However, the data must be interpreted with caution because the pharmacological approach to prevent vascular access thrombosis in hemodialysis is still in its infancy. Overall, the effect of antiplatelet agents on vascular access patency needs further investigation. A prospective randomized controlled trial with larger number of patients is warranted. Currently the National Institutes of Health sponsored Dialysis Access Consortium is conducting an ongoing double-blind multicenter randomized evaluation of clopidogrel in AVF patency.[24]

## Conclusion

Primary AVF failure remains a major problem for hemodialysis patients. Vascular access thrombosis prophylaxis needs to start early in the ESRD patient. Clopidogrel, beginning 7–10 days prior to AVF creation and continuing for six weeks, seems to prevent primary AVF failure with acceptable side effects in selected hemodialysis patients.
